# Auditory brainstem response deficits in learning disorders and developmental language disorder: a systematic review and meta-analysis

**DOI:** 10.1038/s41598-022-20438-7

**Published:** 2022-11-22

**Authors:** Lisa K. Chinn, Marina A. Zhukova, Ryan J. Kroeger, Leandro M. Ledesma, Joslyn E. Cavitt, Elena L. Grigorenko

**Affiliations:** 1 Department of Psychology, University of Houston, Health 1, Room 456, Houston, TX 77204 USA; 2 Laboratory of Translational Sciences of Human Development, Saint Petersburg State University, Saint Petersburg, Russia; 3 Center for Cognitive Sciences, Sirius University, Federal Territory “Sirius” Sochi, Russia; 4 Department of Molecular and Human Genetics, Baylor College of Medicine, Houston, TX USA; 5 Child Study Center and Haskins Laboratories, Yale University, New Haven, CT USA

**Keywords:** Psychology, Human behaviour, Auditory system

## Abstract

Although learning disorders (LD) and developmental language disorder (DLD) can be linked to overlapping psychological and behavioral deficits, such as phonological, morphological, orthographic, semantic, and syntactic deficits, as well as academic (e.g., reading) difficulties, they are currently separate diagnoses in the DSM-5 with explicit phenotypic differences. At a neural level, it is yet to be determined to what extent they have overlapping or distinct signatures. The identification of such neural markers/endophenotypes could be important for the development of physiological diagnostic tools, as well as an understanding of disorders across different dimensions, as recommended by the Research Domain Criteria Initiative (RDoC). The current systematic review and meta-analysis examined whether the two disorders can be differentiated based on the auditory brainstem response (ABR). Even though both diagnoses require hearing problems to be ruled out, a number of articles have demonstrated associations of these disorders with the auditory brainstem response. We demonstrated that both LD and DLD are associated with longer latencies in ABR Waves III, V, and A, as well as reduced amplitude in Waves V and A. However, multilevel subgroup analyses revealed that LD and DLD do not significantly differ for any of these ABR waves. Results suggest that less efficient early auditory processing is a shared mechanism underlying both LD and DLD.

## Introduction

Despite separate diagnostic categories in the DSM-5, learning disorders (LD, exemplified by a reading disability also known as dyslexia) and developmental language disorder (DLD, also sometimes termed specific language impairment or SLI) can have partially overlapping behavioral symptoms and outcomes. For example, according to the DSM-5, both may present with reading and other academic difficulties. However, at a physiological level, we do not yet know to what extent the two disorders have overlapping mechanisms. Common criticisms of the DSM-5 are that it involves non-specific symptoms and often disregards underlying neurobiology^1^. The current systematic review and meta-analysis addresses these issues by examining the existing literature on event-related potentials (ERPs) associated with LD and DLD. The goal is to look for evidence of sensitivity and specificity in a physiological indicator previously shown to be related to both of these disorders: the auditory brainstem response (ABR).

Although researchers have found resting state EEG and event-related potential (ERP) correlates of LD and DLD^2^, these disorders are typically diagnosed behaviorally, without the aid of physiological biomarkers. Our goal of looking for specific neural mechanisms underlying LD and DLD aligns with the Research Domain Criteria Initiative (RDoC) mission to investigate specific biological factors instead of just behavioral manifestations of disorders^3^. Diagnostically sensitive biomarkers would be important for clinical practice, because they could provide an objective diagnostic tool that would be effective at young ages, perhaps before difficulties become apparent in academic settings.

One non-specific criterion for the diagnosis of both LD and DLD is that hearing issues must typically be ruled out (American Psychological Association, APA, 2013). However, a number of studies have examined LD or DLD and observed differences relative to controls in the ABR^4,5^, which measures auditory nerve, cochlear nuclei, and brainstem response to sound and is sometimes used as a hearing test. A variety of ABR waves, such as waves I to V and binomial waveforms, have been examined in LD or DLD individually (e.g., García Planas & García-Camba Vives, et al.^6,7^). Such findings suggest that although a standard hearing test should not detect hearing problems in LD or DLD due to its level of sensitivity and specificity, individuals with LD and DLD may have subtle hearing deficits or deficient early auditory processing relative to typically developing individuals, indicated by effects such as longer latencies in one or more ABR waves. However, it remains an open question whether the ABR can be used to differentiate LD and DLD, or even to distinguish LD and DLD from neurotypicality.

Furthermore, each of the above disorders has primarily been studied in relation to typically developing individuals, not in relation to each other. Research that explicitly examines neural similarities and differences between these disorders using auditory ERPs is limited. Even though a small number of articles directly compare LD and DLD, these articles usually examine higher order cognitive processes and/or ERPs that occur after the ABR^8,9^. The strategy behind the current project was to first perform a systematic review to see whether DLD and LD tend to consistently show effects in the ABR relative to controls, and to then use a meta-analytic approach with a subgroup analysis to compare both disorders to typically developing individuals and LD to DLD. Exploring the specificity in the ABR biomarkers for each of these disorders will contribute to the knowledge of the underlying neurobiological mechanisms of these conditions. By examining the sensitivity and specificity of the ABR we aimed at providing guidance for clinicians and researchers who may benefit from the use of the ABR as a diagnostic tool for LD and/or DLD.

### Auditory brainstem response

The Auditory Brainstem Response (ABR) is an evoked potential used to measure very early auditory processing along structures such as the auditory nerve and cochlear nuclei^10^. Clinical norms have been established for the ABR, and the ABR is used as a diagnostic tool in infants with a known genetic risk for a disorder affecting hearing. It is not, however, currently used to diagnose learning or language disorders. Traditionally, stimuli designed to evoke the ABR consist of rapidly presented clicks around 100 µs in duration with an inter-stimulus interval between 25 and 500 ms^11^. Recently, new methods have also allowed for speech-evoked ABR using various combinations of consonant–vowel syllables as stimuli to detect early language processing^12^. Therefore, our systematic review and, when possible, some of our meta-analyses included information on stimulus type used to evoke the ABR in case one stimulus type could differentiate these two disorders better than another stimulus type. A goal of this project was to investigate the diagnostic potential of the ABR for LD and DLD, for all stimulus types or specifically for clicks or speech.

### Similarities and differences between LD and DLD

As noted above, at the phenotypic level, DLD and LD can involve overlapping non-specific issues, such as deficiencies in certain cognitive processes and academic difficulties. Yet the two disorders are described differently at a phenotypic level and given separate categories in the DSM-5^13^. LD involves a specific disability in reading, writing, and/or math despite normal IQ and adequate educational resources. DLD is described as involving more general difficulties with language acquisition, such as vocabulary and grammar, which may manifest as delays in written, spoken, or sign language.

At a mechanistic level, however, the differences between LD and DLD are less clear. Phonological language processing is typically identified as a common underlying deficit in both disorders^14^, suggesting that differences between LD and DLD may lie elsewhere, such as in earlier sensory processing or higher-order cognitive processes. Some researchers also suggest that DLD and LD are two points on a continuum rather than distinct disorders^15,16^. For instance, some have argued that dyslexia, which is a type of learning disorder with impairments in reading single words^17^, should be considered a developmental language disorder, because one of its underlying mechanisms can be difficulties with the phonological processing part of the language system^18^. In addition to dyslexia, phonological processing difficulties have also been demonstrated to underlie disorders affecting language more broadly than just reading, such as speech sound disorder^19^. Therefore, phonological processing issues alone likely cannot differentiate reading versus language disorders. However, it is unclear whether these disorders have other distinct neural correlates, and whether the magnitude of shared neural processing deficits is the same or different in these two disorders.

Further complicating things, the extent of overlap between LD and DLD may also vary depending on the disorder subtype. In addition to specific impairments in reading, writing, or math, LD subtype may be unspecified or general. Dyslexia has been proposed to have “phonological” and “surface” or “orthographic” subtypes^20,21^, which may involve the auditory or visual systems to differing degrees. For instance, some research suggests that dyslexia is associated with auditory processing deficits^22^, whereas others focus on visual perception^23^ and visual attention deficits^24,25^. Given the overlap between LD and DLD with respect to phonological processing, one might expect altered auditory processing in both LD and DLD, with respect to at least some subtypes of LD. One goal of the current meta-analytic study was to examine consistency in ABR effects across subtypes of these disorders. However, as explained in further detail below, the literature that we identified on the ABR in LD predominately focuses on unspecified LD or LD related to aspects of language, such as reading. Therefore, we could not directly compare subtypes within these disorders in the meta-analysis due to a lack of analyzable sources on some subtypes.

## Method

The current article is part of a larger project on ERPs related to LD, DLD, and intellectual disability (ID). The project topic and the procedures described below were first registered on PROSPERO on May 7, 2020, before searches were conducted. The original protocol was published and assigned number CRD42020188700^26^. A protocol revision was submitted on March 22, 2021 to extend the expected completion date, add a new team member, confirm the addition of a meta-analysis, and suggest that we would publish our findings in parts due to a large number of search results on a wide range of ERP components (over 9 components). The current article focuses on the ABR in LD and DLD only, due to a lack of search results on ID and the ABR. In order to ensure that our broader searches retrieved all articles relevant to the ABR and LD or DLD, and that the results were current, an additional round of searches specific to LD, DLD, and the ABR was conducted on April 21, 2021.

### Similarities and differences between LD and DLD

**Figure 1:**
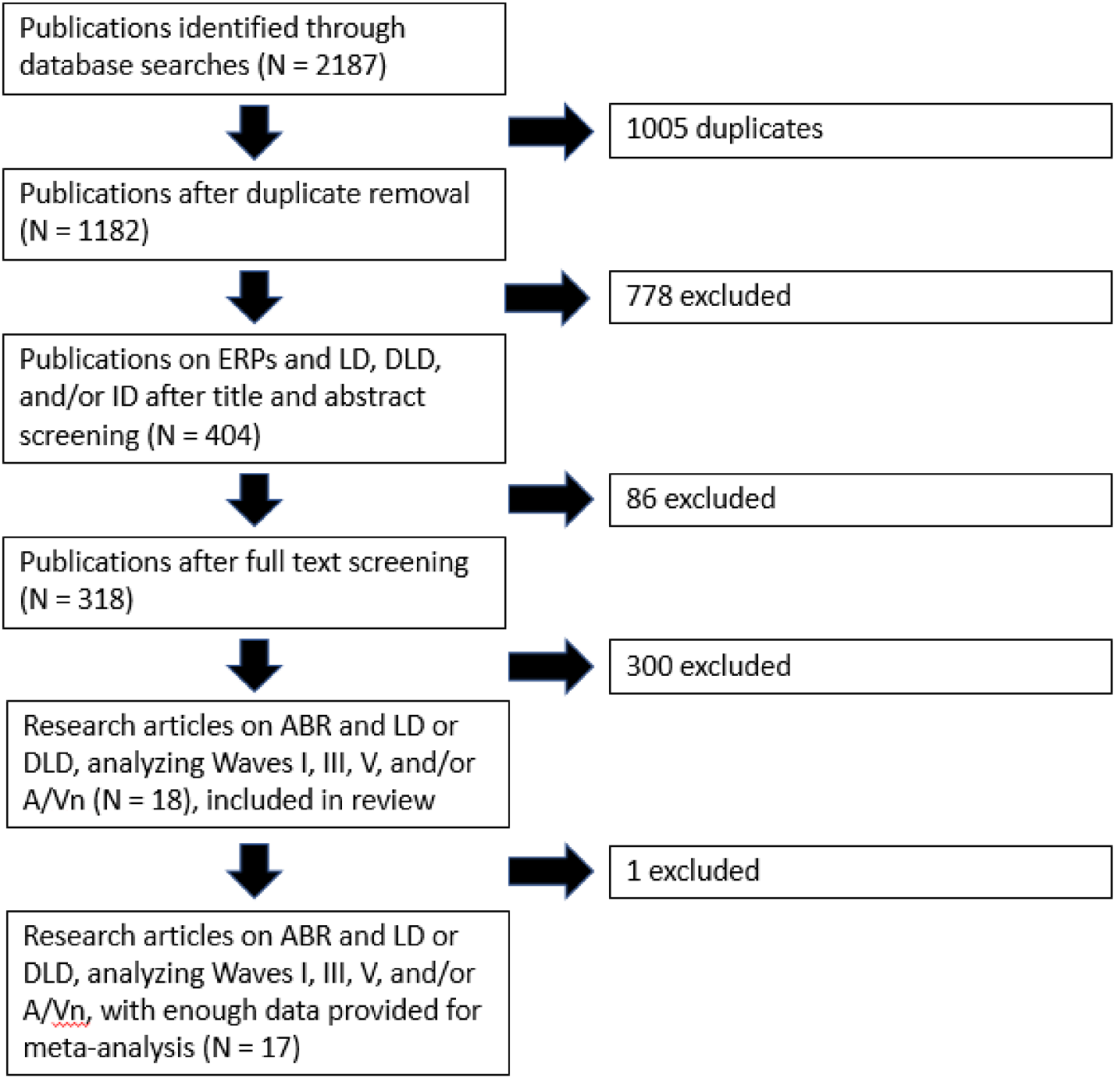
Article selection process with numbers of exclusions.

During preliminary searches of Pubmed and PsychINFO databases, we looked for variations on individual keywords related to ERPs, ID, DLD, and LD using the * “wildcard” operator, as well as terms included in the individual databases’ indexing systems—MeSH terms in Pubmed and thesaurus descriptor terms in PsychINFO. After the relevant indexing terms were identified, the individual keywords related to a concept (e.g., all keywords related to ERPs; see Table 1) were grouped using the OR operator. After each concept (ERPs, ID, DLD, LD) had its search terms grouped using the OR operator, each of the groups of terms related to disorder was combined with the ERP search term group using the AND operator. Searches were conducted in Pubmed and PsycINFO databases on May 16, 2020. Pubmed returned 1340 results, and PsycINFO returned 847 (total = 2187 before removing duplicates). These results were exported to the Mendeley reference manager, where duplicates were removed using the “Merge” feature. After duplicate removal, a total of 1182 articles remained (see Fig. 1).

**Table 1:** Individual search terms searched for variations in both databases, identified Pubmed MeSH terms, and identified PsycINFO descriptor terms.

Theme	Keywords	Pubmed MeSH terms	PsycINFO descriptor terms
ERPs	ERP* "event related" "evoked potential*" “Event-related” “evoked-potential*”	“evoked potential”	N/A
Learning Disabilities	“learning disab*” “learning disorder*” “learning impair*” “learning difficul*” LD	“learning disabilities”	"learning disorders" "learning disabilities"
Intellectual Disabilities	“cognitive disab*” “cognitively disabled” “cognitive disorder*” “intellectual* disab*” “intellectual impair*” “intellectually impaired” “mental retardation” “low* IQ” “low* intelligence” “mental* disab*” “mentally handicapped” ID	“intellectual disability”	"intellectual development disorder" "cognitive impairment"
Developmental Language Disorder	DLD SLI “language impairment*” "language disord*" "language delay*" "delayed language" "communication disorder*"	"specific language disorder" "language development disorders" "communication disorders"	"language disorders" "communication disorders" "specific language impairment" "language delay"

### Article selection

Article titles and abstracts from our search results were imported to the Abstrackr abstract selection tool^27^, which was used to indicate whether they warranted exclusion or further reading for inclusion using the selection criteria outlined below.

#### Selection criteria

The authors identified articles that used ERPs to examine ID, DLD, and/or LD. Specifically, inclusion criteria for the meta-analysis were the following: research conducted with human subjects, ERP measure(s) included, written in English, children (ages 0 to 18) included in the sample, peer reviewed empirical journal articles, and containing participants with ID, DLD and/or LD, including subtypes or major symptoms of these disorders (e.g., dyslexia, low IQ, reading difficulties). Articles on children of parents with one of the above disorders were also included. Articles on auditory processing disorder/central auditory processing disorder were included as DLD and labeled as a subtype in order to allow for comparison to articles that used other terminology such as DLD or SLI. We examined relevant review articles, meta-analyses, and book chapters to inform the introduction and discussion sections of this article, but these types of publications were not included in the systematic review percentages we computed on agreement between results across different studies or in the meta-analyses.

Exclusion criteria were: impairment due to a specific event or non-genetic illness (such as intellectual disability following traumatic brain injury or language impairment following a stroke), a sample consisting of adults only, published before the year 2000, written in a non-English language, a primary focus on hearing loss (such as papers on language ability after a cochlear implant), a focus on disorders affecting speech but not the broader concept of language (such as papers on stuttering or selective mutism), case studies, and papers that did not analyze ERPs. The cutoff of a publication date in 2000 or later was guided by the goal to analyze the most recent studies published in the past 22 years. It might be more difficult to request data from authors for older studies, as some authors have passed away, moved universities without bringing data, or lost access to the data, among other events. Thus, such a cutoff date would reduce publication bias issues, as significant results are more likely to be published and non-significant data/effect sizes are more likely to require a request to the author. To avoid potential redundancy if data from non-peer reviewed student theses, dissertations, or preprints were later published in peer-reviewed journals, non-peer reviewed articles were not included. These selection criteria are outlined in further detail in the published PROSPERO protocol^26^.

#### Selection procedure

Four coders screened article titles and abstracts in Abstrackr in accordance with the inclusion/exclusion criteria outlined above. As per our PROSPERO protocol, all four coders completed a pilot round where they made decisions about ~ 20% of the articles (237/1182 articles). Because inter-rater reliability was moderate for the pilot round (Fleiss Kappa = 0.74), the rest of the articles were double screened. When a conflict occurred, the first and second authors discussed the abstract and decided together whether to include the article.

The next step was to read the full-text articles and extract results from relevant research articles. First, the reader double-checked the full-text version against the inclusion and exclusion criteria described above. After this stage, 318 articles were verified as relevant to our topic and evaluated more carefully by at least one member of a larger research team involved in this analysis and/or the larger set of meta-analyses resulting from the original search; of these, 295 were research articles and 23 were considered “other” (review articles, book chapters, or meta-analyses).

For the purposes of the current paper, only articles that measured amplitude and/or latency for Waves I, III, V, and/or Vn/A of the ABR in LD and/or DLD (N = 18) were examined. Only one article identified in our searches analyzed ABR amplitude or latency in ID (Khaliq et al.^28^), so it was excluded. Analyses on Wave II, Wave IV, inter-peak latency differences, and later ABR waves were excluded from the percentages of articles with significant effects calculated in the systematic review and from the meta-analyses due to a limited number of papers on these measures (e.g., only one DLD paper and no LD papers compared Wave IV amplitude and latency to a control group) and unique, non-comparable analyses in some papers (e.g., different papers analyzing different inter-peak intervals or slopes).

### Second search round

As noted above, an additional round of searches on LD, DLD, and the ABR was conducted on April 21, 2021 using the search terms in Table 2. PsycInfo returned 179 results and PubMed returned 175. After removing duplicates between Pubmed and PsycInfo, and between the current and original search, 144 articles were retained. These articles were exported to Abstrackr and the first and second authors selected or rejected the articles using the criteria above. All articles were double screened. Inclusion and exclusion criteria were the same as for the first round of searches, and no new research articles were identified for inclusion.

**Table 2:** Search terms used in the second search.

Theme	Keywords	Pubmed MeSH Terms	PsycINFO descriptor terms
ABR	ABR “Auditory brainstem response”	NA	N/A
Learning Disabilities	“learning disab*” “learning disorder*” “learning impair*” “learning difficul*” LD	“learning disabilities”	"learning disorders" "learning disabilities"
Developmental Language Disorder	DLD SLI “language impairment*” "language disord*" "language delay*" "delayed language" "communication disorder*"	"specific language disorder" "language development disorders" "communication disorders"	"language disorders" "communication disorders" "specific language impairment" "language delay"

### Information extraction and analyses

For research articles, details on participant groups (diagnoses), stimulus modalities, task conditions, participant ages, sample sizes, ABR components measured, direction of effects, effect sizes, group means, standard deviations, and test statistics were entered into a spreadsheet, when available. For articles examining effects of an intervention or training, only pre-intervention results were included.

## Results

Terminology used varied across the literature (see Supplemental tables). For the purposes of naming in Table 3 and subgroup meta-analyses, groups referred to as specific language impairment (SLI), auditory processing disorder (APD), central auditory processing disorder (CAPD), phonological disorder, language impairment, and language-learning impairment were classified as DLD. Auditory processing disorder (APD) was included as DLD because research has suggested that children with APD have similar profiles to those with DLD and that differential diagnosis might reflect the type of clinician providing the diagnosis more than concrete differences between APD and DLD^29^. LD not otherwise specified, reading problems, and language-based learning disability were classified as LD. Some terms were somewhat ambiguous with respect to classification within our subgroups. Specifically, phonological disorder could in theory be considered a subtype of either dyslexia or a developmental language disorder because both are associated with phonological processing deficits^14,18^. Language-based learning disability could also potentially fit under LD or DLD.

**Table 3:** Percentages of articles with significant effects in analyses of disorder groups relative to controls and percentages of significant effects that occurred in the same direction. One additional article not included in this table (due to measurement differences in quantifying Wave V) presented significantly lower amplitude in the V-Va complex in children with language-based learning problems relative to controls^32^. Note that one article by Purdy and colleagues^33^ included in the LD group Wave V row in this table had participants with both LD and suspected auditory processing disorder. This article found no significant amplitude effect and was the only one with significantly earlier latency for the disorder group relative to controls.

Disorder	Wave	Amplitude			Latency		
		No. of articles (no. of analyses)	Percent analyses with sig. effect	Percent with smaller amplitude in disorder group	No. of articles (no. of analyses)	Percent analy-ses with sig. effect	Percent with later latency in disorder group
LD	I	1(2)	0%	NA	1(2)	50.0%	100%*
DLD	I	1(1)	100%*	100%*	3(4)	25.0%	100%*
LD	III	1(2)	0%	NA	2(3)	33.3%	100%*
DLD	III	0	NA	NA	4(5)	60%	100%
LD	V	4(7)	0%	NA	6(9)	55.56%	60%
DLD	V	5(6)	33.3%	100%	11(21)	57.1%	100%
LD	A/Vn	3(5)	0%	NA	5(7)	71.4%	100%
DLD	A/Vn	2(2)	50%	100%*	7(14)	57.1%	100%

Results Significance and Consistency.

*Indicates only one analysis included in percentage calculation.

### Systematic review

Early ABR components typically occur under 10 ms from stimulus onset and consist of a series of deflections labeled with roman numerals I-V to denote the temporal order in which the peaks occurred^30^. Later ABR components typically begin with Wave A for speech-evoked ABR or Wave Vn for click-evoked ABR^4^, although some refer to the first deflection after Wave V as Wave A for both speech and clicks (e.g., Song et al.^31^). We created a table to aggregate and compare papers that measured amplitude and/or latency for ABR Waves I, III, V, and/or A/Vn. Although Waves A and Vn may have somewhat different physiological bases due to their origins in different types of stimuli, we combined them in one row in Table 3 due to (1) inconsistent naming conventions across papers and (2) effects that tended to occur in similar directions when significant. Results for individual analyses with stimulus type noted are in in the Supplemental Tables. Table 3 contains composite percentages of articles that found significant effects and percentages of effects that occurred in the same direction. Larger tables with results from individual articles are included in the Supplemental Tables. These tables also note any subtypes of LD or DLD returned in the search results (see Supplemental Tables) in order to help clarify whether effects appeared to be general across LD and DLD or specific to certain subtypes or terminologies used to describe the clinical groups in studies included in this paper.

Across all ABR waves examined, the percentage of analyses with significant effects varied substantially, from 0 to 100%. However, when articles did report significant effects, these effects tended to consistently occur in the same direction with respect to disorder versus control groups (see Supplemental Tables for individual article results). Amplitudes tended to be smaller and latencies tended to be later for disorder relative to control groups.

### Meta-analysis

Due to some variation in the subtypes of LD and DLD (described above), the data in forest plots were visually inspected for average differences associated with subtypes of the major disorder categories. However, some disorders had no articles (e.g., dyscalculia) and others had only one (language-based learning difficulties), so subcategories of LD and DLD could not be included as a moderator in the analyses. We also visually inspected for differences associated with stimulus types, when not able to be statistically analyzed. Age could not be included as a covariate in statistical analyses, because many studies had large and partially overlapping age ranges (e.g., 7–15 years, 8–12 years, 6–12 years). However, the most extreme effect sizes or effect sizes that occurred in a different direction than most studies were visually identified in forest plots, and we checked that these did not occur in studies with the youngest or oldest ages.

#### Data analyses

Hedges’ g was computed for each analysis from group sample sizes, means, and standard deviations, when available using the below formula:

$$g = \frac{{M_{1} - M_{2} }}{{SD_{pooled} }}$$

where M_1_ = Group 1 mean, M_2_ = Group 2 mean, and SD_pooled_ = pooled standard deviation.

Pooled standard deviation was calculated as:

$$SD_{pooled} = \sqrt {\frac{{(n_{1} - 1)SD_{1}^{2} + (n_{2} - 1)SD_{2}^{2} }}{{n_{1} + n_{2} - 2}}}$$

When means and standard deviations were not available in an article, Hedges’ g and variance were computed from ANOVA F-values and group sample sizes using the esc_f() function from the esc package^34^ in R^35^. For amplitude analyses, a negative value for Hedges’ g indicated that the disorder group had smaller ABR amplitudes relative to controls. For latency analyses, a positive value for Hedges’ g indicated that the disorder group had longer ABR latencies than neurotypical controls. When results were reported without means and SDs or test statistics, which sometimes occurred for non-significant results, the article’s corresponding author was contacted via email and asked to provide the data within a month. If the author did not respond within two weeks, they were sent a follow-up email. If the author had not responded and more than one month had passed since the initial email, the meta-analysis proceeded without these data. We did not receive additional data for one or more non-significant results from 4 articles. Therefore, although most articles did report the necessary data to include non-significant results in the meta-analysis, our results contain some bias toward significant findings. Additionally, the number of results described in the systematic review is slightly larger than the number included in the meta-analyses, because some non-significant results were reported in the review that did not contain sufficient data for meta-analysis. In addition to a bias toward published papers providing more details on significant versus non-significant results, there may have been a publication bias where studies with statistically significant results were more likely to be accepted for publication than those without significant results.

Meta-analyses were conducted using a multilevel modeling approach in R with the rma.mv() function from the metafor package^36^. Because participants were nested within studies and some papers contained multiple analyses within one study, such as when a single study measured the ABR to more than one stimulus type, and to allow for the possibility that different studies sampled from different populations, a multilevel model was used^37^ with a study variable entered into the model as a random effect^38^ and effect sizes clustered within study. Restricted Maximum Likelihood (REML) estimation was used to estimate parameters^39^. Two or three analyses were run for each measure (amplitude, latency) for each ABR wave analyzed, depending on what was possible given the number of effect sizes. The effect of major disorder category (LD, DLD) was tested as a moderator for all waves, when there were articles on each disorder category and a given wave. For ABR waves with multiple papers that used clicks and speech sounds (Wave V and Wave A/Vn), the effect of stimulus type (clicks, speech sounds, tones) was also analyzed by entering it as a moderator. Whether the overall effect size (for LD and DLD combined) deviated from zero was tested as a main effect for all waves. Although articles with APD/CAPD groups were included in the primary analyses as part of the DLD group (see Sect. 3 above), analyses were also rerun without effect sizes from APD/CAPD studies since there is some debate as to whether APD/CAPD is different from DLD.

Bias was examined using the rma() function to build a funnel model, and then the regtest() function to statistically test funnel plot asymmetry using Egger’s test. These functions are from the metafor package in R^36^. Asymmetry was not statistically significant for amplitude (*p* = 0.12) or latency (*p* = 0.30).

#### Wave I

##### Amplitude

**Figure 2:**
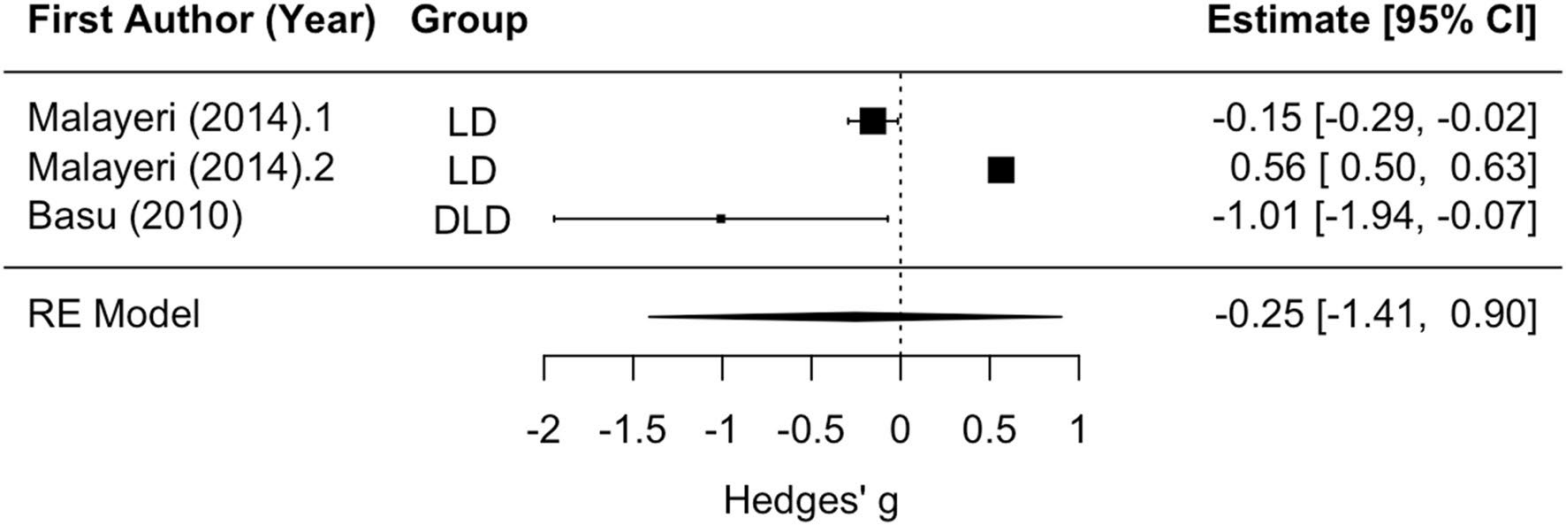
Wave I amplitude forest plot. Positive values for Hedges’ g indicate larger amplitude for the disorder group relative to controls. Rows with decimal points after the article year indicate multiple analyses from the same paper, and correspond to the row order of the analysis in the systematic review Supplement A.

Only two articles analyzed here examined Wave I amplitude—one on DLD^40^ and one on LD/reading problems^4^. The DLD article reported significantly smaller Wave I amplitude for the DLD group relative to controls, and the LD article did not find statistically significant effects of LD status on Wave I amplitude for clicks or speech sounds (see Supplement A). Subgroup analysis did not reveal a significant difference between LD and DLD. However, this result should be interpreted with caution due to the small number of articles included in this analysis and significant residual heterogeneity (Qe_(1)_ = 85.24, *p* < 0.001; see Fig. 2). Additionally, the LD article had a speech stimulus condition with a positive effect size, versus click stimuli analyses which had negative effect sizes in the two articles. The DLD article did not have a speech stimulus condition. Therefore, further research on speech versus click sounds on Wave I amplitude in each disorder may be warranted. With LD and DLD and clicks and speech analyzed together, the overall mean effect size for disorder groups compared to controls did not significantly differ from 0. Neither of the articles that measured Wave I amplitude contained a group with APD or CAPD, so analyses were not rerun to exclude these effect sizes.

##### Latency

**Figure 3:**
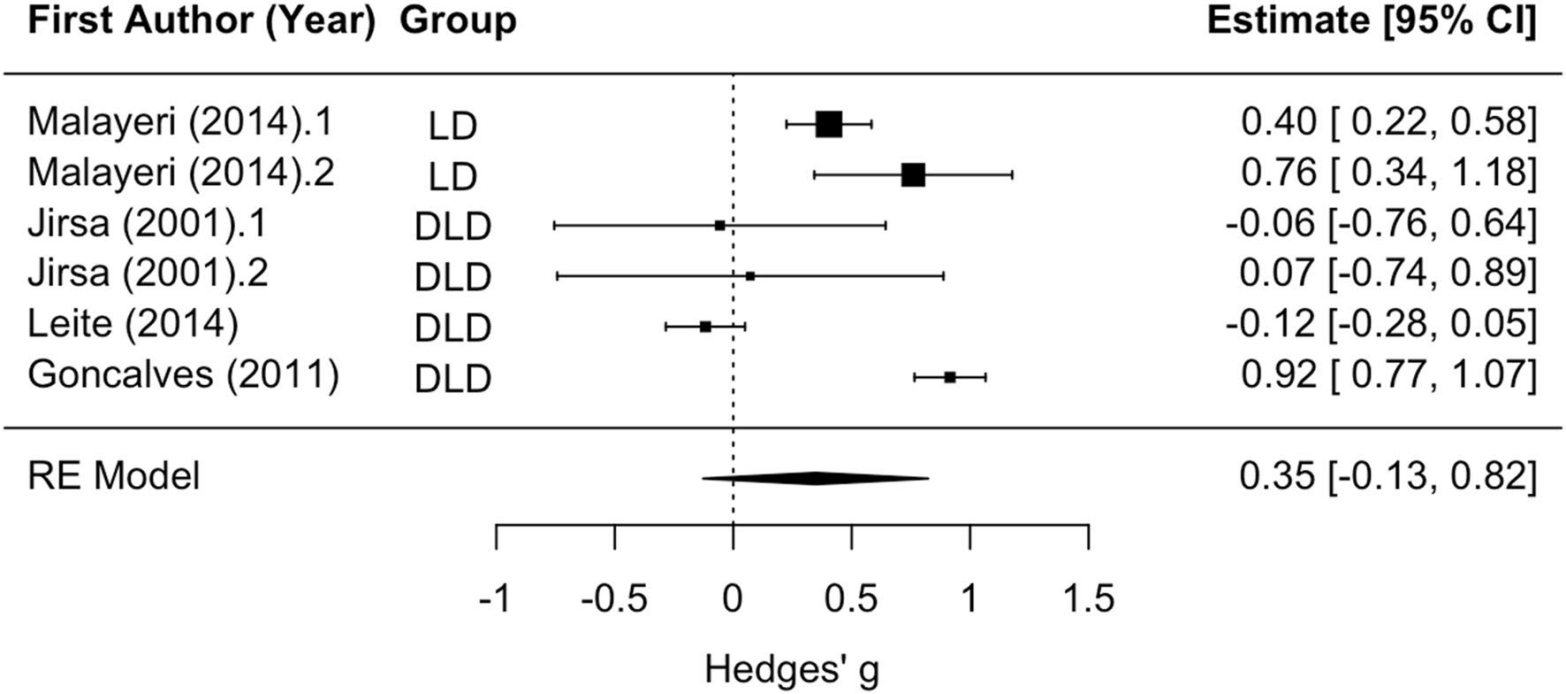
Wave I Latency forest plot. Positive values for Hedges’ g indicate longer latency for the disorder group relative to controls. Rows with decimal points after the article year indicate multiple analyses from the same paper, and correspond to the row order of the analysis in the systematic review table in Supplement A.

With respect to Wave I latency, there was no significant subgroup effect of disorder type (LD, DLD). Without subgroups included in the model the mean effect size for analyses of the disorder groups versus controls was not significantly different than zero. Significant residual heterogeneity was also present (Q_(5)_ = 86.45, *p* < 0.001). Visual inspection (see Fig. 3 and Supplement A) revealed that inconsistencies in effects did not seem to be due to factors such as participant age ranges, disorder subtypes, or stimulus types (clicks vs. speech, left vs. right ear). For instance Leite et al.^41^ did not find a significant effect but Gonçalves et al.^5^ did, although both studies contained participants with phonological disorder with largely overlapping age ranges of 8–11 and 7–11 years, respectively. Confidence intervals for the same samples in Malayeri and colleagues^4^ were largely overlapping for click and speech sounds, and in Jirsa et al.^42^ for right and left ear measures. Therefore, all stimulus conditions were combined in analyses above, and it was not possible to test for differences between LD and DLD with respect to effects of clicks versus speech sounds or left versus right ear. With effect sizes on APD or CAPD versus controls removed from the analyses (Jirsa et al.^42^), the results were the same (no significant difference between LD and DLD, no significant effect of disorder groups and controls, and significant heterogeneity present).

#### Wave III

##### Amplitude

Only one DLD article^4^ provided data or test statistics on Wave III amplitude, so this measure was not meta-analyzed.

##### Latency

**Figure 4:**
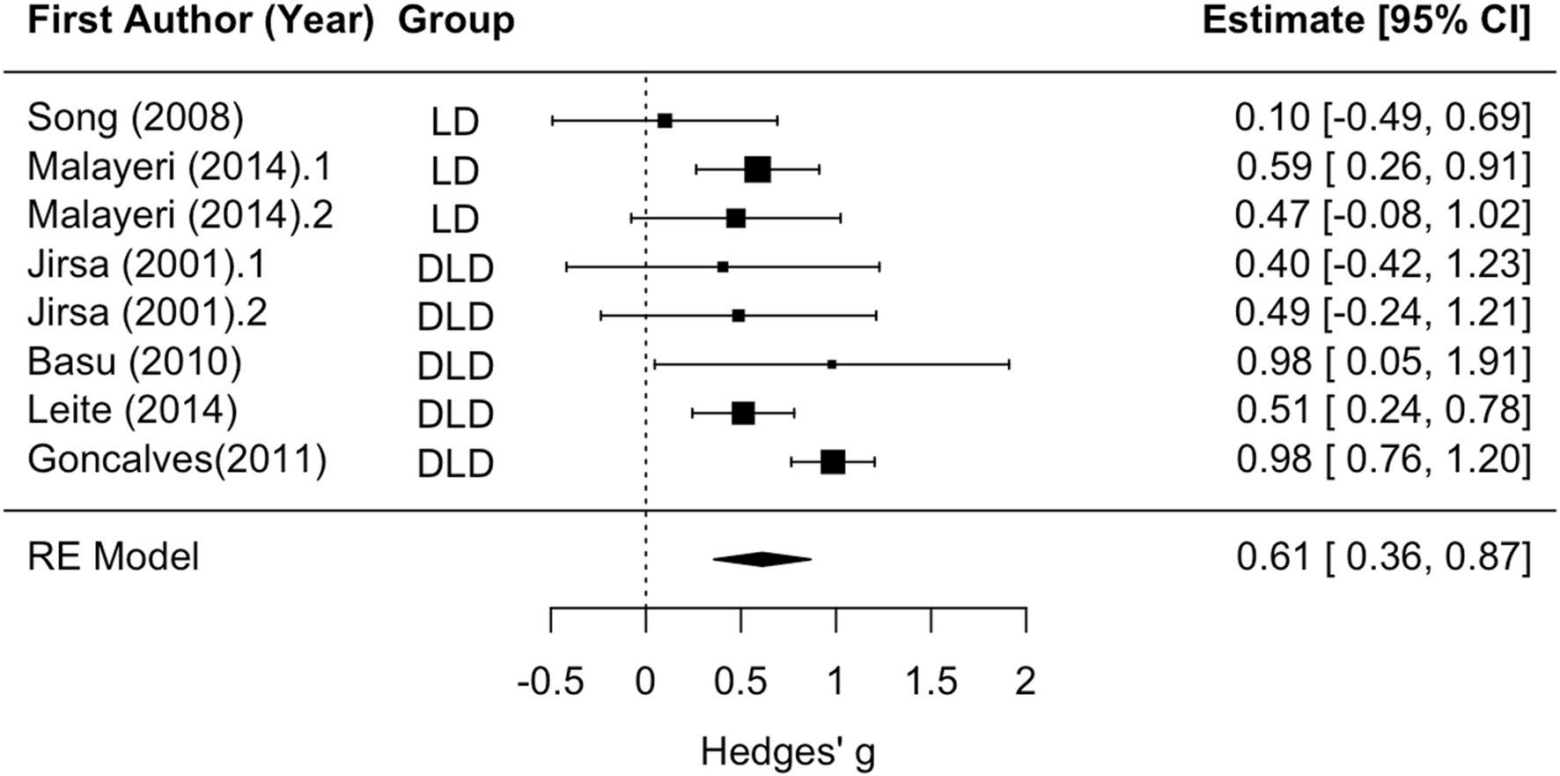
Wave III latency forest plot. Positive values for Hedges’ g indicate longer latency for the disorder group relative to controls. Rows with decimal points after the article year indicate multiple analyses from the same paper, and correspond to the row order of the analyses in the systematic review table in Supplement B.

LD and DLD subgroup analysis revealed no significant difference between the two groups with respect to Wave III latency. The mean effect size for analyses of the disorder groups versus controls was significantly different than zero (*z* = 4.72, *p* < 0.001; see Fig. 4), such that Wave III latency tended to be longer in the disorders relative to controls (mean Hedges’ g = 0.61). Residual heterogeneity was statistically significant (Q_(7)_ = 14.53, *p* < 0.05). As with Wave I, only one article on one disorder (LD) analyzed speech sounds^4^ and only one analyzed each ear separately, so it was not possible to test whether stimulus type or presentation method (speech vs. click sounds, left vs. right ear) had different effects in LD versus DLD. However, the analyses on different stimulus types in those two papers had largely overlapping confidence intervals, so here we decided it was acceptable to combine all stimulus types in analyses on group effects on latency. With effect sizes on APD or CAPD versus controls removed from the analyses (Jirsa et al.^42^), the results were approximately the same. LD and DLD did not significantly differ. The mean effect size (mean Hedges’ g = 0.63) for disorder versus control groups was significantly different from zero (*z* = 4.25, *p* < 0.001), and significant heterogeneity was present (Q_(5)_ = 13.75, *p* < 0.05).

#### Wave V

##### Amplitude

**Figure 5:**
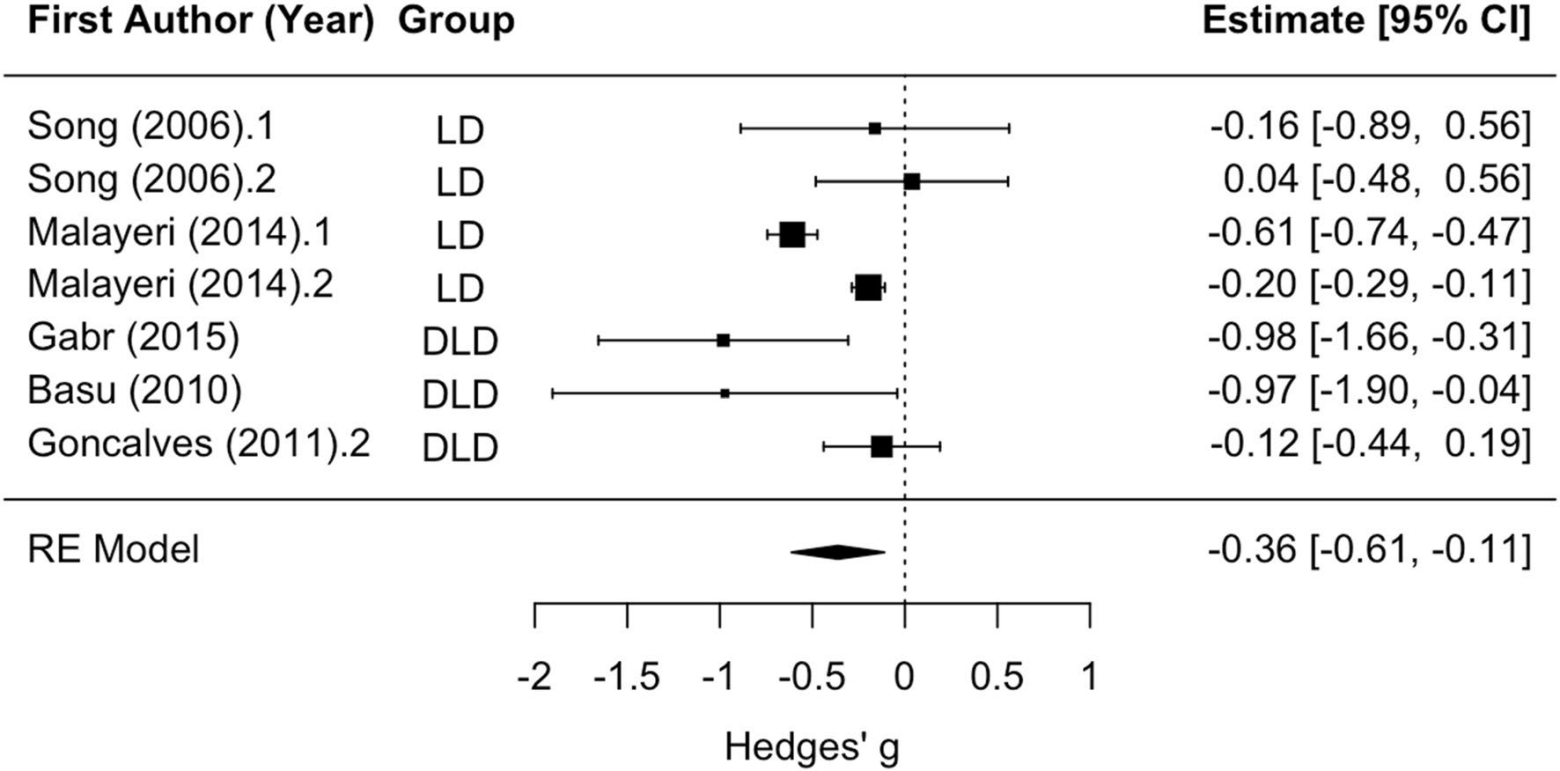
Wave V Overall Amplitude effect forest plot. Negative values of Hedges’ g indicate lower amplitude in the disorder group relative to the control group. Rows with decimal points after the article year indicate multiple analyses from the same paper, and correspond to the row order of the analysis in the systematic review table in Supplement C.

Subgroup analysis revealed no significant difference between LD and DLD groups with respect to Wave V amplitude. The mean effect size for analyses of the disorder groups versus controls was significantly different than zero (z =  − 2.83, *p* < 0.01; see Fig. 5), such that amplitude tended to be smaller in the disorder groups relative to controls (mean Hedges’ g =  − 0.36). Statistically significant residual heterogeneity was present (Q_(6)_ = 33.71, *p* < 0.001). None of the articles that examined Wave V amplitude contained a group with APD or CAPD, so analyses were not rerun to exclude these effect sizes.

##### Latency

**Figure 6:**
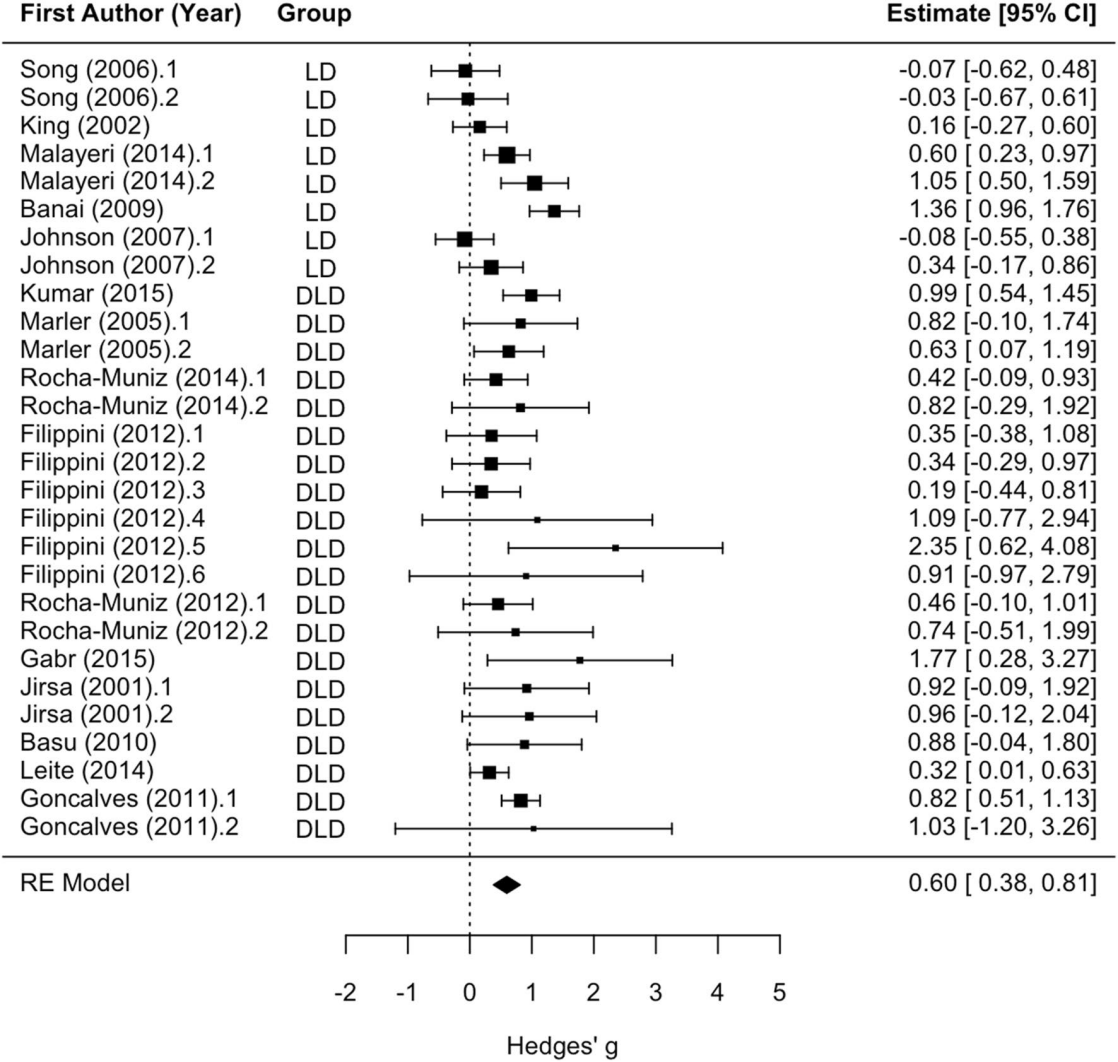
Wave V Overall latency effect forest plot. Positive values of Hedges’ g indicate longer latency for the disorder group relative to controls. Rows with decimal points after the article year indicate multiple analyses from the same paper, and correspond to the row order of the analysis in the systematic review table in Supplement C.

Moderator analyses revealed no significant difference between LD and DLD groups and no significant effect of stimulus type (clicks, speech sounds, tones) or the Group x Stimulus Type interaction for Wave V latency. One paper by Purdy and colleagues^33^ was excluded from the moderator analyses including disorder (LD, DLD) because its participants did not fit cleanly into LD or DLD subgroups due to a diagnosis of LD with suspected auditory processing disorder. The main effect of disorder groups versus controls was statistically significant (z = 4.54, *p* < 0.001; see Fig. 6), such that disorder groups tended to have later Wave V latency relative to controls (mean Hedges’ g = 0.55). Statistically significant residual heterogeneity was present (Q_(27)_ = 69.60, *p* < 0.001). With effects sizes that contained an APD/CAPD group removed from the analyses (Purdy et al.^33^; Kumar et al.^43^; one analysis from Rocha-Munis et al.^44^; two analyses from Filippini et al.^45^; one analysis from Rocha-Muniz et al.^46^; Jirsa et al.^42^), the group and stimulus type effects were still not statistically significant. The main effect of disorder groups versus controls was statistically significant (mean Hedges’ g = 0.58, z = 4.44, *p* < 0.001). Statistically significant residual heterogeneity was present (Q_(20)_ = 53.09, *p* < 0.001).

#### Wave A

##### Amplitude

**Figure 7:**
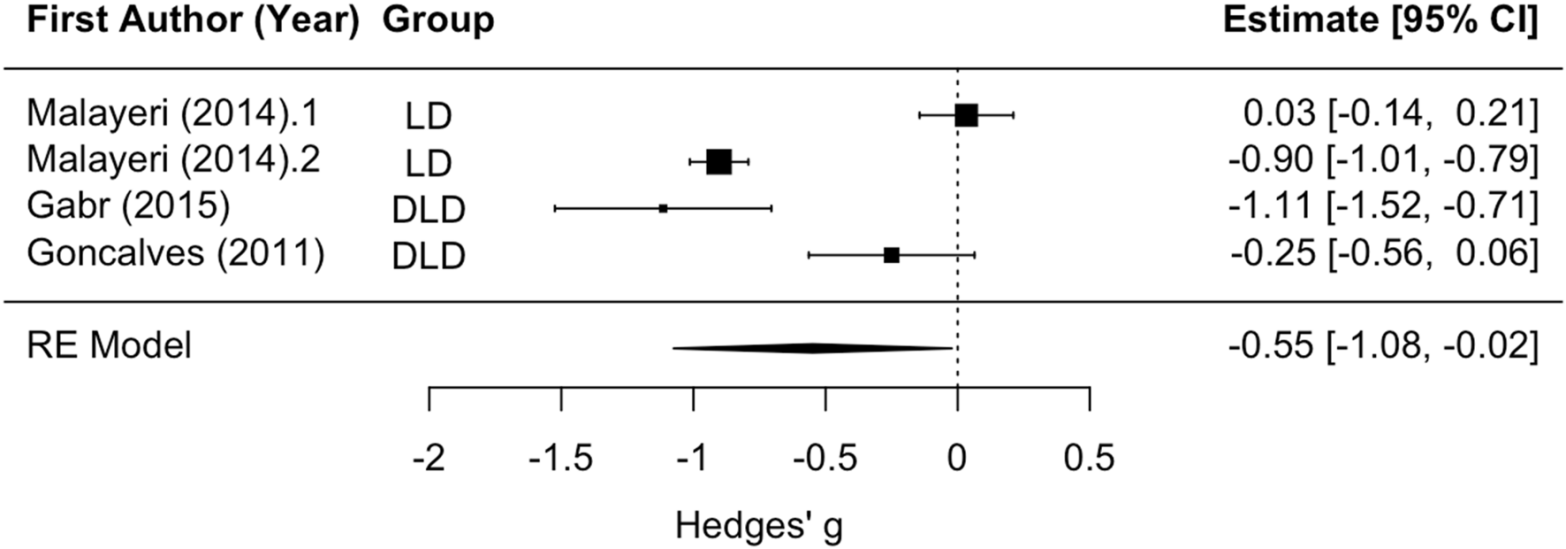
Wave A/Vn Amplitude forest plot. Negative values of Hedges’ g indicate lower amplitude in the disorder group relative to the control group. Although subgroup and analyses of stimulus type were not possible due to the limited number of effect sizes in this analysis, the two statistically significant effects were from articles with two different stimulus types (Malayeri (2014).2 – speech, Gabr (2005) – clicks), so differences between papers were not likely due a difference between speech and non-speech stimuli. Rows with decimal points after the article year indicate multiple analyses from the same paper, and correspond to the row order of the analysis in the systematic review table in Supplement D.

There was no statistically significant subgroup effect of disorder type (LD vs. DLD) on Wave A amplitude. The mean effect size for analyses of disorder groups versus controls was significantly different than zero (z =  − 2.04, *p* < 0.05), such that disorder groups tended to have reduced amplitude relative to controls (mean Hedges’ g =  − 0.65; see Fig. 7). However, this effect should be interpreted with caution due to a limited number of studies (n = 3) that analyzed Wave A amplitude and the presence of statistically significant residual heterogeneity (Q_(3)_ = 88.10, *p* < 0.001). None of the articles that examined Wave A amplitude contained a group with APD or CAPD, so analyses were not rerun to exclude these effect sizes.

##### Latency

**Figure 8:**
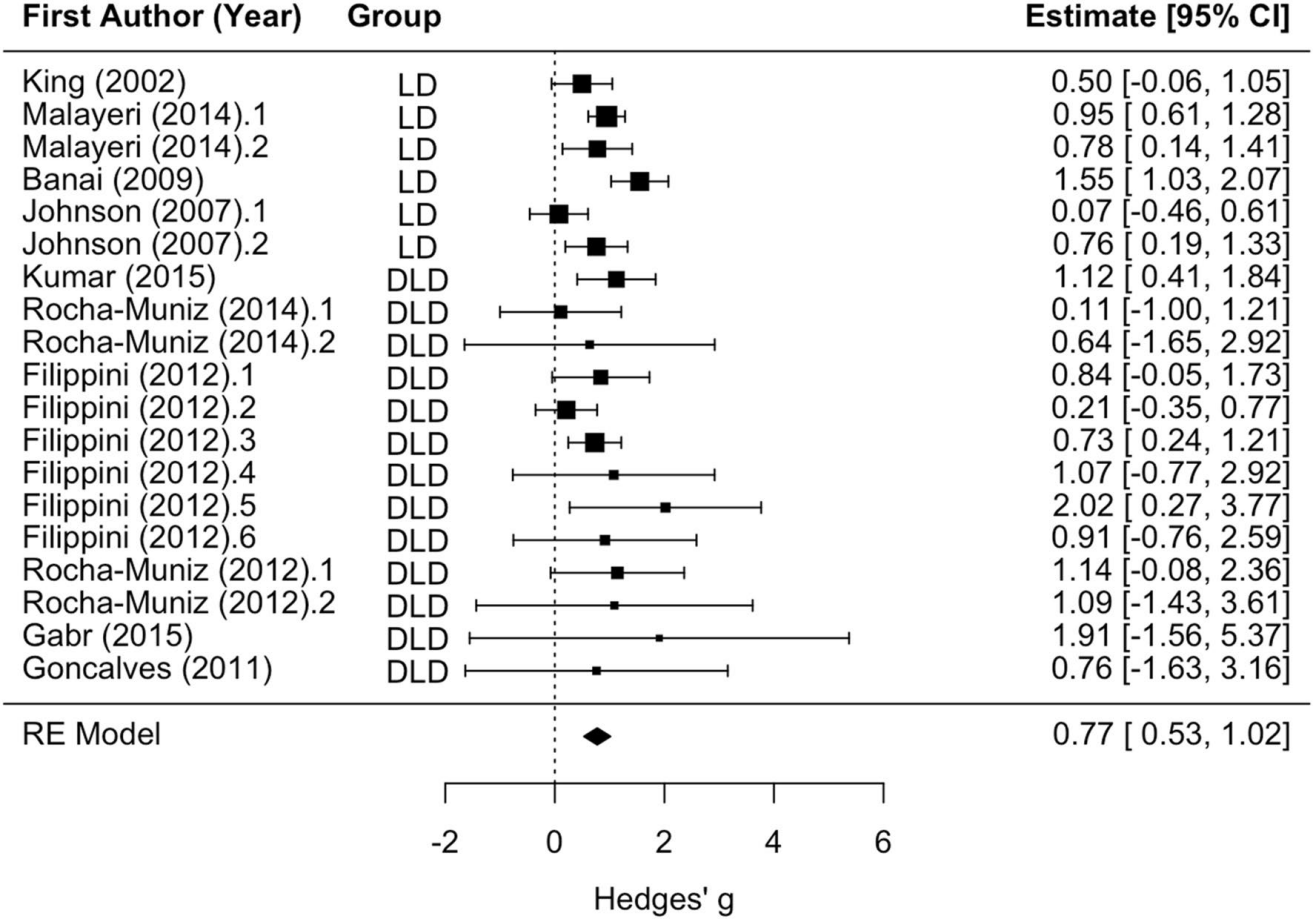
Wave A/Vn latency forest plot. Positive values of Hedges’ g indicate longer latency for the disorder group relative to controls. Rows with decimal points after the article year indicate multiple analyses from the same paper, and correspond to the row order of the analysis in the systematic review table in Supplement D.

Moderator analyses revealed no significant difference between LD and DLD groups and no significant effect of stimulus type (clicks, speech sounds, tones) or the Group x Stimulus Type interaction for Wave A/Vn latency. The main effect of disorder groups versus controls was statistically significant (z = 6.26, *p* < 0.001; see Fig. 8), such that disorder groups tended to have later Wave A/Vn latency relative to controls (mean Hedges’ g = 0.77). Residual heterogeneity was not statistically significant (*p* = 0.09). With effect sizes on analyses of APD/CAPD groups removed (Kumar et al.^43^; one analysis from Rocha-Muniz et al.^44^; two analyses from Filippini et al.^45^; one analysis from Rocha-Munis et al.^46^), moderator analyses still revealed no significant difference between LD and DLD groups and no significant effect of stimulus type (clicks, speech sounds, tones) or the Group x Stimulus Type interaction for Wave A/Vn latency. The main effect of disorder groups versus controls was statistically significant (mean Hedges’ g = 0.75, z = 5.08, *p* < 0.001). Statistically significant residual heterogeneity was present (Q_(13)_ = 23.47, *p* < 0.05).

## Discussion

Our goal was to examine the literature on the ABR in LD and DLD to assess whether the ABR shows sensitivity and specificity with respect to these disorders. Because articles directly comparing LD and DLD are limited, we used multilevel modeling to fill this gap and statistically compare the two disorders. Our systematic review and meta-analyses revealed that ABR latencies were longer in Waves III, V, and A/Vn in both DLD and LD relative to typically developing individuals. Amplitudes were also smaller in Waves V and A/Vn in the disorder groups relative to controls. These results indicate that two disorders may overlap in their underlying mechanisms with respect to early auditory processing, although additional studies would be needed to assess similarities and differences for the two disorders with respect to all stimulus types (clicks, speech sounds, tones) in all ABR Waves (e.g., Wave I or Wave VI).

Although meta-analyses revealed consistently longer latencies and reduced amplitudes in a number of ABR waves in both LD and DLD, these effects were not always large or statistically significant in individual studies. Additionally, for most measures, residual variance significantly differed across studies. Therefore, the ABR may be sensitive to both LD and DLD, but the small and/or variable effects in some studies may indicate that the diagnostic potential of the ABR is limited. Additionally, most subgroup analyses revealed that DLD and LD were not significantly different from each other, suggesting a potential lack of specificity in the ABR. This result aligns with other recent work that has found a lack of specificity in other disorders such as speech impairment and ASD^47^. The only potential exception in our study was Wave I amplitude, where one paper found significantly reduced amplitude in DLD (SLI) and another found no significant amplitude effects for LD (reading problems). However, due to the limited number of studies that examined Wave I amplitude, additional work is needed to determine whether this measure can distinguish LD from DLD. Additionally, the DLD article that analyzed Wave I only included click sounds, so further work would be needed to determine whether the two groups differ in their Wave I responses to speech sounds.

Although we did not specifically include auditory processing disorder (APD) or central auditory processing disorder (CAPD) as search terms, our searches returned some articles that contained a clinical group with APD/CAPD. These groupings occurred because some researchers consider DLD and APD/CAPD to be the same^29^ although one article that we analyzed found different ABR latency results for an APD group relative to controls than an SLI group relative to controls^46^. We did not design the current study with a prediction related to similarities or differences between APD/CAPD and DLD/SLI. Nonetheless, in order to rule out the possibility that our results were driven by APD/CAPD participants that may have differed from DLD participants, we performed all analyses to exclude effect sizes from APD/CAPD groups. The results did not differ without these effect sizes.

A possible explanation for the current results is that a range of issues with language, including specific impairments in reading or writing (LD) and impairments in generalized language ability (DLD), could all be driven by less efficient auditory processing. Indeed, many studies have uncovered auditory origins of both these disorders^48,49^. Although these disorders may manifest with somewhat different behavioral phenotypes, as detected by individuals and their teachers or parents, results indicate that LD and DLD have at least partially overlapping physiological mechanisms. Therefore, despite separate diagnostic categories in the DSM-5, they may not be fully distinct from each other at early levels of processing and may both benefit from interventions that target early sensory processing.

Another possibility is that auditory processing issues are secondary to another deficit or multiple deficits in one or both of these disorders. For instance, some researchers have suggested that dyslexia is rooted in visual attention deficits and is not primarily an issue of phonological processing^25^. In theory, attention and sensory processing could be linked, as attentional issues could cause reduced sensory processing of stimuli that are not fully attended to. However, the auditory brainstem response occurs at an early stage of sensory processing and tends to be the same in awake and unconscious people^50^. Therefore, the current findings suggest that attentional differences or other higher-order cognitive processes requiring consciousness are unlikely to be the only mechanism(s) underlying LD and DLD.

A limitation of the current project is that our searches did not return any articles on the ABR waves we analyzed and dyscalculia, a subtype of LD with deficits in math ability^51^. The searches also did not return articles on specific subtypes of dyslexia that have been proposed, such as phonological and surface dyslexia, or enough articles that looked at Wave VI or binomial waves to meta-analyze all possible ABR measures examined in the literature (e.g., García Planas and García-Camba Vives et al.^6,7^). Therefore, it is unclear whether the findings extend to all subtypes of LD, how different subtypes compare to each other, or how LD and DLD compare on all possible ABR measures. Furthermore, some articles analyzed in this meta-analysis had differing inclusion/exclusion criteria for their disorder groups. For example, some relied on parent-reported case history and verified their language difficulties with language tests (e.g., Basu et al.^40^). Others relied on referrals through a clinic or lab to obtain a clinical sample (e.g., Leite et al^41^). Although we generally felt that inclusion/exclusion criteria for the articles in our search results were justifiable, a limitation of the current study is that we could not be sure that individuals classified as having a disorder in one study would have been classified the same in all other studies.

Some articles, such as a study that found a link between genes associated with dyslexia and ABR consistency^52^, could not be included in our meta-analyses because they used measures or data analysis techniques that differed from other articles included here. Furthermore, the literature on differences between APD and DLD is inconsistent. Here, due to sample size constraints and overlap between the two diagnoses^29^, we included APD as a subtype of DLD. However, some researchers believe these disorders differ, and one manuscript analyzed here compared SLI to APD and found some differences in measures such as balance of Wave A^53^. Additional studies containing both LD and DLD groups and their various subtypes within the same study would help, above and beyond the current paper’s mixed effects models, to be sure that the two groups do not differ on some ABR indicator in response to a specific stimulus type. A final limitation is that, due to a small number of search results on the ABR and LD or DLD (n = 18) and smaller numbers of analyses on each ABR wave within those articles, it is possible that some of our statistical analyses, especially those involving interaction terms, were under-powered. As more research accumulates on this topic in the future, researchers might want to attempt to replicate the current meta-analysis.

Our ongoing work on a larger project will systematically review and perform meta-analyses on the literature on ERPs associated with later auditory processing and higher-order cognitive processes, including the MMN, P300, and N400 in LD, DLD, and ID. The presence of studies that use visual paradigms to test some of these later components will help us address whether issues in these disorders are specific to the auditory modality, or if other sensory domains and/or higher-order cognitive processes are implicated. Some studies also use multimodal tasks^54^, which may enable the examination of sensory integration as a mechanism behind neurodevelopmental disorders affecting language. Results from these future meta-analyses will add to the current study’s findings that both LD and DLD involve early auditory processing deficits but that the ABR does not have good specificity for distinguishing between the two disorders.

## Supplementary Information

Supplementary Information 1.

Supplementary Information 2.

## Data Availability

Data analyzed in this meta-analysis are included in the supplementary information files.
