# Supplementary material for: Auditory brainstem response deficits in learning disorders and developmental language disorder: a systematic review and meta-analysis
Source: Sci Rep. 2022 Nov 22;12:20124. doi: 10.1038/s41598-022-20438-7 (PMC9684495; doi:10.1038/s41598-022-20438-7)
Supplement: Supplementary file 1 — Supplementary Information 1. [file 41598_2022_20438_MOESM1_ESM.docx]

# Auditory brainstem response deficits in learning disorders and developmental language disorder: A systematic review and meta-analysis

# Lisa K. Chinn, Marina A. Zhukova, Ryan J. Kroeger, Leandro M. Ledesma, Joslyn E. Cavitt, Elena L. Grigorenko

**Supplemental Tables**

***Wave I***

| Authors, year | Disorder | Subtype | Task/Stimuli | Parameters | Age range | Total N (Disorder N) | Amplitude Effect | Latency Effect | Other |
| --- | --- | --- | --- | --- | --- | --- | --- | --- | --- |
| Song, Banai, & Kraus, 2008 | LD | Not specified | Speech | Five-formant speech syllable /da/, 40 ms in duration, initial 10-ms burst centered around the beginning frequencies of formants 3–5 in the range of 2580 to 4500 Hz | 8-12 yrs | 183  (93) | - | ns. | Wave I was only detected in 59/90 controls and 44/93 LD participants. |
| Malayeri, Lotfi, Moossavi, Rostami, & Faghihzadeh, 2014 | LD | Reading difficulties, could also have attention deficit disorder (ADD) | Clicks | 100 μs clicks presented at a rate of 11.1/s, 80 dB SPL, 30-3000 Hz band-pass filtering | 8-12 yrs | 83  (49) | ns. | ns. |  |
|  | LD | Reading difficulties, could also have attention deficit disorder (ADD) | Speech | 40-ms /da/, 10.9/sec, 80 dB SPL | 8-12 yrs | 83  (49) | ns. | → |  |
| Gonçalves, Wertzner, Samelli, & Matas,  2011 | DLD | Phonological disorder | Clicks | 100 µs clicks presented at a rate of 19.1 clicks/s, 80 dBnHL, 150 to 3,000 Hz filter | 7-11 yrs | 36  (18) | - | → |  |
| Jirsa, 2001 | DLD | APD | Clicks (right ear) | 100-µs clicks delivered through the insert phones at 70 dB nHL at a rate of 909.1/sec, not occurring simultaneously in both ears | 9-13 yrs | 67  (37)  6 APD participants also had ADHD. 19 were receiving treatment at school for speech and language. | - | ns. | Fewer subjects with disorder had an identifiable Wave I relative to controls |
|  | DLD | APD | Clicks (left ear) | 100-µs clicks delivered through the insert phones at 70 dB nHL at a rate of 909.1/sec, not occurring simultaneously in both ears | 9-13 yrs | 67  (37)  6 APD participants also had ADHD. 19 were receiving treatment at school for speech and language. | - | ns. | Fewer subjects with disorder had identifiable Wave I relative to controls |
| Basu,  Krishnan, & Weber-Fox,  2010 | DLD | SLI | Clicks | 100 μsec click stimuli of alternating polarity presented at 70 dB nHL for repetition rates of 11.1/s, 21.1/s, 51.1/s and 71.1/s | 4-11 yrs | 20  (10) | ↓ | ns. | Also found differences in the frequency following response (FFR) to upswept and downswept tones, such that the SLI group had worse tracking of the frequency rate at higher sweep rates. |
| Leite, Wertzner, Gonçalves, Magliaro, & Matas,  2014 | DLD | Phonological disorder | Clicks | 19.1 clicks/s, 0.1 ms duration, 12 dB/octave filter slope, 100-3000 Hz band pass filter, 2000 sweeps, 80 dBnHL | 8-11 yrs | 47  (23) | - | ns. | No left-right difference. Also found increased Wave I-III distance. |

*Supplement A.* Wave I results. When an article has more than one analysis per disorder subtype and component, the title is left blank in immediately following rows on the same article. ns. = effect not statistically significant. - = effect not analyzed. ↑ = larger effect associated with disorder group relative to controls. ↓ = smaller effect associated with disorder group relative to controls. → = later effect associated with disorder group relative to controls. ← = earlier effect associated with disorder group relative to controls.

***Wave III***

| Author last names, year | Disorder | Subtype | Task/Stimuli | Parameters | Age range | Total N (Disorder N) | Amplitude Effect | Latency Effect | Other |
| --- | --- | --- | --- | --- | --- | --- | --- | --- | --- |
| Song, Banai, & Kraus,  2008 | LD | Not specified | Clicks | Five-formant speech syllable /da/, 40 ms in duration, initial 10-ms burst centered around the beginning frequencies of formants 3–5 in the range of 2580 to 4500 Hz | 8-12 yrs | 183  (93) in entire study  174(88) with detectable Wave III | - | ns. | 5 LD and 4 controls were missing Wave III. This difference was ns. In controls Wave III was correlated with a rostral brainstem timing score. Ns. correlation in LD group. |
| Malayeri, Lotfi, Moossavi, Rostami, & Faghihzadeh,  2014 | LD | Reading difficulties, could also have attention deficit disorder (ADD) | Clicks | 100 μs clicks presented at a rate of 11.1/sec, 80 dB SPL, 30-3000 Hz band-pass filtering | 8-12 yrs | 83  (49) | ns. | → |  |
|  | LD | Reading difficulties, could also have attention deficit disorder (ADD) | Speech | 40-ms /da/, 10.9/sec, 80 dB SPL | 8-12 yrs | 83  (49) | ns. | ns. |  |
| Song, Banai, Russo, & Kraus, 2006 | LD | Not specified “learning problem” (LP). Children grouped by normal or abnormal responses to /da/ stimulus | Speech | 11.1 Hz presentation rate; 40 ms five-formant /da/ with initial 10ms burst around the beginning frequencies of formants 3-5 in 2580 to 4500 range | 8-12 yrs | 234  (119), 183 with normal /da/ response, 51 abnormal | - | - | More controls vs. LP group had a normal response to /da/ (97 vs. 86) and fewer controls vs. LP group had an abnormal response (18 vs. 33). *The abnormal /da/ response group had a Wave III latency delay for /da/. |
| Leite, Wertzner, Gonçalves, Leite Magliaro, & Matas,  2014 | DLD | Phonological disorders | Clicks | 19.1 clicks/s, 0.1 ms duration, 12 dB/octave filter slope, 100-3000 Hz band pass filter, 2000 sweeps, 80 dBnHL | 8-11 yrs | 47  (23) | - | → | Also found increased Wave I-III distance. No left-right difference. |
| Gonçalves,  Wertzner, Samelli, Gentile, &  Matas,  2011 | DLD | Phonological disorder | Clicks | 100 µs clicks presented at a rate of 19.1 clicks/s, 80 dBnHL, 150 to 3,000 Hz filter | 7-11 | 36  (18) | - | → | Also found ns. amplitude and latency effects in Waves C & F. |
| Jirsa,  2001 | DLD | APD | Clicks (right ear) | 100-µs clicks delivered through the insert phones at 70 dB nHL at a rate of 909.1/sec, not occurring simultaneously in both ears | 9-13 yrs | 67  (37)  6 APD participants also had ADHD. 19 were receiving treatment at school for speech and language. | - | ns. | Fewer subjects with DLD had identifiable Wave III than controls |
|  | DLD | APD | Clicks (left ear) | 100-µs clicks delivered through the insert phones at 70 dB nHL at a rate of 909.1/sec, not occurring simultaneously in both ears | 9-13 yrs | 67  (37)  6 APD participants also had ADHD. 19 were receiving treatment at school for speech and language. | - | ns. | Fewer subjects with DLD had identifiable Wave III relative to controls |
| Basu, Krishnan, Weber-Fox,  2010 | DLD | SLI | Clicks | 100 μsec click stimuli of alternating polarity presented at 70 dB nHL for repetition rates of 11.1/s, 21.1/s, 51.1/s and 71.1/s | 4-11 yrs | 20  (10) | - | → | Also found differences in the frequency following response (FFR) to upswept and downswept tones, such that the SLI group had worse tracking of the frequency rate at higher sweep rates. |

*Supplement B.* Wave III results. When an article has more than one analysis per disorder subtype and component, the title is left blank in immediately following rows on the same article. Ns. = effect not statistically significant. - = effect not analyzed. ↑ = larger effect associated with disorder group relative to controls. ↓ = smaller effect associated with disorder group relative to controls. → = later effect associated with disorder group relative to controls. ← = earlier effect associated with disorder group relative to controls. *This analysis was not included in the meta-analysis or Table 3, because participants were grouped for this analysis based on ABR response, not LD diagnosis status.

***Wave V***

| Author last names, year | Disorder | Subtype | Task/Stimuli | Parameters | Age range | Total N (Disorder N) | Amplitude Effect | Latency Effect | Other/Notes |
| --- | --- | --- | --- | --- | --- | --- | --- | --- | --- |
| Malayeri, Lotfi, Moossavi, Rostami, Faghihz adeh,  2014 | LD | Reading difficulties, could also have attention deficit disorder (ADD) | Clicks | 100 μs clicks presented at a rate of 11.1/sec, 80 dB SPL, 30-3000 Hz band-pass filtering | 8-12 yrs | 83  (49) | ns. | → | Also found longer V-Vn and V-A inter-peak latency in LD. Children with learning problems had no correlation between ABRs for speech and clicks, but controls did. |
|  | LD | Reading difficulties, could also have attention deficit disorder (ADD) | Speech | 40-ms /da/, 10.9/sec, 80 dB SPL | 8-12 yrs | 83  (49) | ns. | → |  |
| Song, Banai, Russo, & Kraus, 2006 | LD | Not specified “learning problem” | Clicks in quiet | 100 μs clicks presented at a rate of 31.1 Hz | 8-12 yrs | 234  (119) | ns. | ns. | There was an overall effect of quiet versus noise such that noise introduced a later latency and smaller amplitude in both groups. |
|  | LD | Not specified “learning problem” | Clicks in noise | 100 μs clicks presented at a rate of 31.1 Hz | 8-12 yrs | 234  (119) | ns. | ns. |  |
|  | LD | Not specified “learning problem” (LP). Children grouped by normal or abnormal responses to /da/ stimulus | Speech | 11.1 Hz presentation rate; 40 ms five-formant /da/ with initial 10ms burst of formants 3-5 in 2580 to 4500 range | 8-12 yrs | 234  (119), 183 with normal /da/ response, 51 abnormal | - | - | More controls vs. LP group had a normal response to /da/ (97 vs. 86) and fewer controls vs. LP group had an abnormal response (18 vs. 33). The abnormal group had a significant delay between waves V and A. |
| King,  Warrier, Hayes, & Kraus,  2002 | LD | Not specified | Clicks | 100 μs clicks presented at a rate of 31.1/s, 68 dB HL | 8-12 yrs | 87  (54) | - | ns. |  |
| Banai  Hornickel, Skoe, Nicol, Zecker, & Kraus,  2009 | LD | Not specified | Speech | 40-ms synthesized /da/, fundamental frequency that linearly rose from 103 to 125 Hz, voicing beginning at 5 ms, onset noise burst during the first 10 ms, presented to right ear at 10.9 Hz through insert earphones, 80.3 dB SPL | 7-15 yrs | 63  (25) | - | → |  |
| Johnson,  Nicol, Zecker, & Kraus, 2007 | LD | Language-based learning problems  (Good Backward Masking Score Clinical Group) | Speech | 40 msec /da/ synthesized at a 10 kHz sampling rate, presented to right ear at 80 dB SPL | 8-12 yrs | 54(30) | ns. | ns. |  |
|  | LD | Language-based learning problems  (Bad Backward Masking Score Clinical Group) | Speech | 40 msec /da/ synthesized at a 10 kHz sampling rate, presented to right ear at 80 dB SPL | 8-12 yrs | 40(16) | ns. | ns. |  |
| Purdy, 2002 | LD + possible APD | Diagnosed with LD, and suspected of APD | Clicks | 100-μsec rarefaction clicks presented at 8.7/sec and 70 dB nHL | 7 to 11 yrs | 20(10) | ns. | ← | Also found a shorter wave III-V interval for the LD group. Middle latency response (Na) was later in the LD group relative to controls, and Nb was less negative for LD. |
| Jirsa,  2001 | DLD | APD | Clicks (right ear) | 100-sec clicks delivered through the insert phones at 70 dB nHL at a rate of 909.1/sec, not occurring simultaneously in both ears | 9-13 yrs | 67  (37)  6 APD participants also had ADHD. 19 were receiving treatment at school for speech and language. | - | → |  |
|  | DLD | APD | Clicks (left ear) | 100-sec clicks delivered through the insert phones at 70 dB nHL at a rate of 909.1/sec, not occurring simultaneously in both ears | 9-13 yrs | 67  (37)  6 APD participants also had ADHD. 19 were receiving treatment at school for speech and language. | - | → |  |
| Kumar & Singh, 2015 | DLD | APD (at risk) | Speech  (both ears) | 40 ms synthesized /da/, 80 dB nHL, presented monaurally at a rate of 10.9 Hz | 8-12 yrs | 30  (15) | - | → | The authors analyzed left and right ears separately but found no left/right difference, so they combined the two ears in the stats. |
| Rocha-Muniz,  Befi-Lopes, & Schochat,  2012 | DLD | APD | Speech | 40 ms /da/ syllable synthesized at a 10 kHz, voicing beginning at 5 ms, onset release burst during the first 10 ms | 5 yrs | 57  (18) | - | ns. | Also found delayed latency in some analyses of other ERPs, such as Waves C, E, F, and O. |
|  | DLD | SLI | Speech | 40 ms /da/ syllable synthesized at a 10 kHz, voicing beginning at 5 ms, onset release burst during the first 10 ms | 5 yrs | 57  (21) | - | → |  |
| Rocha-Muniz, Befi-Lopes, & Schochat,  2014 | DLD | SLI | Speech | 5-formant, 40 ms, speech syllable /da/ synthesized at 10 Hz, presented to right ear at 10.9 Hz rate | 6-12 yrs | 75  (25 with SLI, 25 with APD) | - | → | A classification model could classify SLI, APD, and typical development (TD) correctly greater than half the time. Also found delayed latency in Waves D, E, & F were later for SLI compared with TD and APD. Better sensitivity was observed for SLI than APD. |
|  | DLD | APD | Speech | 5-formant, 40 ms, speech syllable /da/ synthesized at 10 Hz, presented to right ear at 10.9 Hz rate | 6-12 yrs | 75  (25 with APD, 25 with SLI) | - | → |  |
| Basu, Krishnan, & Weber-Fox,  2010 | DLD | SLI | Clicks | 100 μsec click stimuli of alternating polarity presented at 70 dB nHL for repetition rates of 11.1/s, 21.1/s, 51.1/s and 71.1/s | 4-11 yrs | 20  (10) | ↓ | Ns. (trending later) | Also found differences in the frequency following response (FFR) to upswept and downswept tones, such that the SLI group had worse tracking of the frequency rate at higher sweep rates. |
| Filippini,  Befi-Lopes, & Schochat,  2012 | DLD | SLI (group a) | Speech (silence) | 40-ms synthesized /da/, presented through earplugs monaurally to the right ear at alternating polarities and 80 dB nHL, collected in silence | 7-13 yrs | 13(6) | - | ns. | Groups a & b later received different interventions, and were therefore analyzed separately, but effects reported here are pre-test only. Also analyzed Wave C and found ns. group amplitude and latency effects. |
|  | DLD | SLI (group b) | Speech (silence) | 40-ms synthesized /da/, presented at 10.9 Hz through earplugs monaurally to the right ear at alternating polarities and 80 dB nHL, collected in silence |  | 15(8) | - | ns. |  |
|  | DLD | APD | Speech (silence) | 40-ms synthesized /da/, presented at 10.9 Hz through earplugs monaurally to the right ear at alternating polarities and 80 dB nHL, collected in silence |  | 16(9) | - | ns. |  |
|  | DLD | SLI (group a) | Speech (background noise) | 40-ms synthesized /da/, presented at 10.9 Hz through earplugs monaurally to the right ear at alternating polarities and 80 dB nHL, collected in ipsilateral white noise (+5 dB SNR) |  | 13(6) | - | → |  |
|  | DLD | SLI (group b) | Speech (background noise) | 40-ms synthesized /da/, presented at 10.9 Hz through earplugs monaurally to the right ear at alternating polarities and 80 dB nHL, collected in ipsilateral white noise (+5 dB SNR) |  | 15(8) | - | ns. |  |
|  | DLD | APD | Speech (background noise) | 40-ms synthesized /da/, presented at 10.9 Hz through earplugs monaurally to the right ear at alternating polarities and 80 dB nHL, collected in ipsilateral white noise (+5 dB SNR) |  | 16(9) | - | ns. |  |
| Gabr & Darwish, 2015 | DLD | SLI | Clicks | 206 ms /da/ presented to both ears | 3-7 yrs | 40(20) | ↓ | → | Also found reduced amplitude and delayed latency in Waves C, D, E, F, & O. |
| Marler & Champlin, 2005 | DLD | Language learning impairment | Pure tone, backward masking condition | Alternating polarity 1-kHz tone with an overall duration of 10 ms, narrow-band masking noise from 0.6 kHz to 1.4 kHz with a 150 ms duration, presented to right ear with insert earphone | 8-10yrs | 20  (10) | ns. | → |  |
|  |  |  | Pure-tone, no masking condition | Alternating polarity 1-kHz tone with an overall duration of 10 ms, presented to right ear with insert earphone | 8-10yrs | 20  (10) | ns. | ns. |  |
| Wible,  Nicol, & Kraus,  2005 | DLD | Language-based learning problems (LP) | Speech | Synthesized speech sounds that varied in the onset frequency of the third formant, spanning from /da/ (2580 Hz) to /ga/ (2180 Hz), presented to the right ear via insert headphones at 80 dB SPL | 11 yrs | 20  (11) | ns. | ns. | Duration of the wave V–V_n_ complex was longer in LP children. |
| Leite,  Wertzner, Gonçalves, Leite Magliaro, Gentile Matas,  2014 | DLD | Phonological disorder | Clicks | 19.1 clicks/s, 0.1 ms duration, 12 dB/octave filter slope, 100-3000 Hz band pass filter, 2000 sweeps, 80 dBnHL | 8-11 yrs | 47  (23) | - | → | No left-right difference. Also found significantly longer Wave I-V difference in disorder group. Ns. difference in Wave III-V. |
| Gonçalves,  Wertzner, Samelli, Matas,  2011 | DLD | Phonological disorder | Clicks | 100 µs clicks presented at a rate of 19.1 clicks/s, 80 dBnHL, 150 to 3,000 Hz filter | 7-11 | 36  (18) | - | → | Also found ns. amplitude and latency effects in Waves C & F. |
|  | DLD | Phonological disorder | Speech | 40 ms of five‐formant speech syllable /da/, 80 dBA, stimulus rate of 11.1/s, right ear presentation | 7-11 | 36  (18) | ns. | → |  |

*Supplement C.* Wave V results. When an article has more than one analysis per disorder subtype and component, the title is left blank in immediately following rows on the same article. Ns. = effect not statistically significant. - = effect not analyzed. ↑ = larger effect associated with disorder group relative to controls. ↓ = smaller effect associated with disorder group relative to controls. → = later effect associated with disorder group relative to controls. ← = earlier effect associated with disorder group relative to controls.

***Waves A and Vn***

| Author last names, year | Disorder | Subtype | Wave | Task/Stimuli | Parameters | Age range | Total N (Disorder N) | Amplitude Effect | Latency Effect | Other |
| --- | --- | --- | --- | --- | --- | --- | --- | --- | --- | --- |
| Malayeri, Lotfi, Abdollah Moossavi, Rostami, & Faghihzadeh,  2014 | LD | Reading difficulties, could also have attention deficit disorder (ADD) | Wave Vn | Clicks | 100 μs clicks presented at a rate of 11.1/sec, 80 dB SPL, 30-3000 Hz band-pass filtering | 8-12 yrs | 83  (49) | ns. | → | Also found longer V-Vn and V-A inter-peak latency. Children with learning problems had no correlation between ABRs for speech and clicks, but controls did. |
|  | LD | Reading difficulties, could also have attention deficit disorder (ADD) | Wave A | Speech | 40-ms /da/, 10.9/sec, 80 dB SPL | 8-12 yrs | 83  (49) | ns. | → |  |
| King,  Warrier, Hayes, & Kraus,  2002 | LD | Not specified | Wave A | Speech | 40 ms of a five-formant synthetic speech syllable /da/, 5 ms rise and fall, right ear presentation at 80 dB SPL, rate of 11.1/s | 8-12 yrs | 87  (54) | - | → |  |
| Song,  Banai, Russo, Kraus, & 2006 | LD | Not specified | Wave A | Clicks | 100 μs clicks presented at a rate of 31.1 Hz | 8-12 yrs | 234  (119) | ns. | ns. |  |
|  | LD | Not specified | Wave A | Speech | 11.1 Hz presentation rate; 40 ms five-formant /da/ with initial 10ms burst of formants 3-5 in 2580 to 4500 range | 8-12 yrs | 234  (119) | - | - | More controls vs. LP group had a normal response to /da/ (97 vs. 86) and fewer controls vs. LP group had an abnormal response (18 vs. 33). The abnormal group had a significant delay between waves V and A. |
| Banai,  Hornickel, Skoe, Nicol, Zecker, Kraus,  2009 | LD | Not specified | Wave A | Speech | 40-ms synthesized /da/, fundamental frequency that linearly rose from 103 to 125 Hz, voicing beginning at 5 ms, onset noise burst during the first 10 ms, presented to right ear at 10.9 Hz through insert earphones, 80.3 dB SPL | 7-15 yrs | 63  (25) | - | → |  |
| Johnson,  Nicol, Zecker, & Kraus, 2007 | LD | Language based learning disability (good backward masking score) | Wave A | Speech | 40 msec /da/ synthesized at a 10 kHz sampling rate, presented to right ear at 80 dB SPL | 8-12 yrs | 54(30) | ns. | ns. |  |
|  | LD | Language based learning disability (bad backward masking score) | Wave A | Speech | 40 msec /da/ synthesized at a 10 kHz sampling rate, presented to right ear at 80 dB SPL | 8-12 yrs | 40(16) | ns. | → |  |
| Wible,  Nicol, & Kraus,  2005 | DLD | Language-based learning problems (LP) | Wave Vn | Speech | Synthesized speech sounds that varied in the onset frequency of the third formant, spanning from /da/ (2580 Hz) to /ga/ (2180 Hz), presented to the right ear via insert headphones at 80 dB SPL | 11 yrs | 20  (11) | ns. | ns. | Duration of the wave V–V_n_ complex was longer in LP children. |
| Gonçalves, Wertzner, Samelli, & Matas,  2011 | DLD | Phonological disorder | Wave A | Speech | 40 ms of five‐formant speech syllable /da/, 80 dBA, stimulus rate of 11.1/s, right ear presentation | 7-11 | 36  (18) | - | → |  |
| Kumar & Kumar Singh,  2015 | DLD | Auditory processing disorder (at risk) | Wave A | Speech  (both ears) | 40 ms synthesized /da/, 80 dB nHL, presented monaurally at a rate of 10.9 Hz | 8-12 yrs | 30  (15) | - | → | The authors analyzed left and right ears separately but found no left/right difference, so they combined the two ears in the stats. |
| Rocha-Muniz  ,Befi-Lopes, & Schochat,  2014 | DLD | SLI | Wave A | Speech | 5-formant, 40 ms, speech syllable /da/ synthesized at 10 Hz, presented to right ear at 10.9 Hz rate | 6-12 yrs | 75  (25) | - | → | See Appendix C. |
|  | DLD | APD | Wave A | Speech | 5-formant, 40 ms, speech syllable /da/ synthesized at 10 Hz, presented to right ear at 10.9 Hz rate | 6-12 yrs | 75  (25) | - | → |  |
| Gabr & Darwish, 2015 | DLD | SLI | Wave A | Clicks | 206 ms /da/ presented to both ears | 3-7 yrs | 40(20) | ↓ | → | Also found reduced amplitude and delayed latency in Waves C, D, E, F, & O. |
| Rocha-Muniz,  Befi-Lopes, & Schochat,  2012 | DLD | APD | Wave A | Speech | 40 ms /da/ syllable synthesized at a 10 kHz, voicing beginning at 5 ms, onset release burst during the first 10 ms | 6-12 yrs | 36(18) | - | → | Also found delayed latency in some analyses of other ERPs, such as C, E, F, and O. |
|  | DLD | LI | Wave A | Speech | 40 ms /da/ syllable synthesized at a 10 kHz, voicing beginning at 5 ms, onset release burst during the first 10 ms | 6-12 yrs | 39(21) | - | → |  |
| Filippini, Befi-Lopes, & Schochat,  2012 | DLD | SLI (group a) | Wave A | Speech (silence) | 40-ms synthesized /da/, presented through earplugs monaurally to the right ear at alternating polarities and 80 dB nHL, collected in silence | 7-13 yrs | 13(6) | - | ns. | Groups a & b later received different interventions, and were therefore analyzed separately, but effects reported here are pre-test only. Also analyzed Wave C and found ns. group amplitude and latency effects. |
|  | DLD | SLI (group b) | Wave A | Speech (silence) | 40-ms synthesized /da/, presented through earplugs monaurally to the right ear at alternating polarities and 80 dB nHL, collected in silence | 7-13 yrs | 15(8) | - | ns. |  |
|  | DLD | APD | Wave A | Speech (silence) | 40-ms synthesized /da/, presented through earplugs monaurally to the right ear at alternating polarities and 80 dB nHL, collected in silence | 7-13 yrs | 16(9) | - | ns. |  |
|  | DLD | SLI (group a) | Wave A | Speech (background noise) | 40-ms synthesized /da/, presented at 10.9 Hz through earplugs monaurally to the right ear at alternating polarities and 80 dB nHL, collected in ipsilateral white noise (+5 dB SNR) | 7-13 yrs | 13(6) | - | → |  |
|  | DLD | SLI (group b) | Wave A | Speech (background noise) | 40-ms synthesized /da/, presented at 10.9 Hz through earplugs monaurally to the right ear at alternating polarities and 80 dB nHL, collected in ipsilateral white noise (+5 dB SNR) | 7-13 yrs | 15(8) | - | ns. |  |
|  | DLD | APD | Wave A | Speech (background noise) | 40-ms synthesized /da/, presented at 10.9 Hz through earplugs monaurally to the right ear at alternating polarities and 80 dB nHL, collected in ipsilateral white noise (+5 dB SNR) | 7-13 yrs | 16(9) | - | ns. |  |

*Supplement D.* Wave A/Vn results. When an article has more than one analysis per disorder subtype and component, the title is left blank in immediately following rows on the same article. Ns. = effect not statistically significant. - = effect not analyzed. ↑ = larger effect associated with disorder group relative to controls. ↓ = smaller effect associated with disorder group relative to controls. → = later effect associated with disorder group relative to controls. ← = earlier effect associated with disorder group relative to controls.
